# Why is SARS-CoV-2 infection milder among children?

**DOI:** 10.6061/clinics/2020/e1947

**Published:** 2020-05-11

**Authors:** Patricia Palmeira, José Alexandre M Barbuto, Clovis Artur A Silva, Magda Carneiro-Sampaio

**Affiliations:** I Laboratorio de Investigacao Medica (LIM-36), Departamento de Pediatria, Hospital das Clinicas HCFMUSP, Faculdade de Medicina, Universidade de Sao Paulo, Sao Paulo, SP, BR; II Departamento Imunologia, Instituto de Ciencias Biomedicas, Universidade de Sao Paulo, Sao Paulo, SP, BR; III Departamento de Pediatria, Faculdade de Medicina FMUSP, Universidade de Sao Paulo, Sao Paulo, SP, BR

Acute respiratory viral diseases, like most other infectious diseases, affect mainly infants and young children, who present with 6-8 episodes per year and usually develop more severe manifestations than in adults. It is well-documented that innate antiviral immune responses in early life are characterized by lower type I interferon (IFN) responses 1 and reduced natural killer cell activity, which despite their higher numbers exhibit lower cytotoxic capacity and reduced number of cytoplasmic granules and degranulation ability 2. Additionally, lower cytotoxic T lymphocyte activity and numbers of effector and memory CD8^+^ T cells have also been described at younger ages 3.

Most data indicate a significant role for innate immunity and T-cell cytotoxicity in the control of viral infections. Surprisingly, however, as seen in the severe acute respiratory syndrome-related coronavirus (SARS-CoV) 4 and Middle East respiratory syndrome-related coronavirus (MERS) 5 outbreaks, the current SARS-CoV-2 pandemic shows low morbidity and near-absent mortality in previously healthy children. On February 28, 2020, in one of the first publications on the clinical features of SARS-CoV-2 infection, Guan et al. 6 analyzed 1,099 laboratory-confirmed patients from Wuhan, China. Among these, only nine were under 14 years (0.9%) and only one had a severe course. Shortly thereafter, a review of 72,314 cases, conducted by the Chinese National Center for Disease Control and Prevention, showed that less than 1% of cases were in children under 10 years of age 7. Similarly, reports from Italy, Brazil, and the USA confirm a lower incidence of serious infections among younger individuals 8-10.

In late March 2020, the Chinese Center for Disease Control and Prevention reported the epidemiological characteristics of a nationwide case series of 2,143 pediatric patients (<18 years old) with COVID-19, including 731 laboratory-confirmed cases and 1,412 suspected patients 11. Among the confirmed cases, 12.9% were asymptomatic, and symptomatic disease was mild in 43.1%, moderate in 41%, and severe in 2.5% of cases. Only 0.4% (3 patients) were classified as critical. Considering the available data for the whole series, the most severe cases were more frequent among those under 5 years old. Clinical data for 171 confirmed cases (1 day to 15 years old) from the Wuhan Children's Hospital were described in more detail 12. Like in adults, there was a predominance of males (60.8%), and the clinical manifestations were quite similar: fever was present in 41.5% of the children and adolescents at any time during the illness, and other common features were cough and pharyngeal erythema. Pneumonia was diagnosed in 111 patients (64.9%), 33 (19.3%) presented only upper respiratory tract manifestations, and 27 (15.8%) had asymptomatic infection. Bilateral ground-glass opacities were the most common radiologic finding, observed in 32.7% of the cases. Three patients required intensive care support and invasive mechanical ventilation (1.75%). These patients had co-existing morbidities (hydronephrosis, leukemia in maintenance chemotherapy, and bowel intussusception), and the only death in the series occurred in a 10-month-old patient with intussusception.

As with SARS-CoV, COVID-19 is believed to be initiated by the binding of the SARS-CoV-2 envelope-anchored spike protein to the outer surface of the angiotensin-converting enzyme 2 (ACE2) catalytic domain 13, promoting endocytosis where viral and host membranes fuse and consequent entry of the virus into the host cell. Angiotensin-converting enzyme (ACE) and its later described homolog ACE2 are critical proteases for regulating the renin-angiotensin system (RAS), exerting opposite roles. Whereas ACE generates angiotensin II, promoting vasoconstriction, ACE2 cleaves angiotensin II to generate Ang1–7, which acts as a negative regulator and exerts an antihypertensive effect 14,15. Zhao et al. 16 reported that ACE2 pulmonary expression is concentrated mainly in type II alveolar cells, which express many other genes that could favor viral replication, thus offering an explanation for the severe alveolar damage associated with SARS-CoV-2 infection. However, one should remember that, in addition to the lung, ACE2 is highly expressed in the kidneys, heart, and testes and is expressed at a lower level in the colon and liver 17. Furthermore, ACE2 may not be the only cellular receptor for the virus. Infection of T lymphocytes, which express very low levels of ACE2, has been described and attributed to the binding of the virus spike protein to CD147, another cell surface molecule 18.

Nevertheless, considering ACE2 as the main gate for infection, the first hypothesis for the diminished susceptibility of children to SARS-CoV-2 suggests a different ACE2 configuration, concentration, or binding capacity or a less harmful alveolar epithelial cell response to ACE2 in children when compared with that in adults 19. Although attractive and supported by observations that some comorbidities associated with a more severe evolution of COVID-19 may be also associated with modifications of ACE2 expression 20-23, the role of ACE2 modulation in this infection is far from clear. Reports suggesting a protective role against severe COVID-19 by increased ACE2 expression are paralleled by others that indicate otherwise 24. In agreement with the hypothesis that ACE2 expression levels have a significant role in acute respiratory distress syndrome (ARDS), which also occurs in COVID-19, an experimental mouse model of H5N1 virus-induced lung injury and death showed ACE2 downregulation following infection 25. In this context, however, one should add a confounding observation: arterial hypertension, a condition that is associated with modified ACE2 expression 26 and was one of the main comorbidities in the Chinese population with severe COVID-19, is barely present among the first North American series reported by the CDC 27. However, it is possible that the increased representation of male individuals among patients with confirmed COVID-19 might be because of decreased ACE2 expression caused by testosterone in contrast to the enhancement caused by estrogens 28,29, a phenomenon that, although not explored in children, might take part in their relative resistance.

Finally, a recently released news report of a fatal case of COVID-19 in a 3-month-old infant with Bartter's syndrome has indicated that ACE2 does have a significant role in COVID-19. This is an interesting example of how rare genetic disorders may contribute to understanding the pathophysiology of common diseases: patients affected with this autosomal recessive tubulopathy have increased ACE2 levels and elevated renin and aldosterone levels 30. However, how these factors actually interact in the case of a SARS-CoV-2 infection remains to be determined.

The aforementioned suggestion by Fang and Luo 19 that the intracellular response induced by ACE2 is different in children than in adults, especially in the elderly, leads us to another hypothesis. In animal models, as age increases, there is a shift in the balance between the pulmonary RAS enzymes, ACE and ACE2. As ACE levels increase, so do the angiotensin II levels, leading to more intense inflammation and increased lung injury 31. Although the same ACE/ACE2 imbalance was not observed in humans in a later study by the same group 32, the incidence, susceptibility, course, and mortality from ARDS do tend to increase progressively with age 33-35. It is well-known that aging is associated with a process called immunosenescence, that is, the decline in the efficiency of the immune systems with age 36. Increasing age is associated with increased neutrophil elastase activity, primary granule release, inaccurate migration, and increased oxidative stress, leading to a state of systemic inflammation 37 with impaired repair mechanisms, thus contributing to exaggerated responses and tissue injury in the elderly 35. In contrast, could the relative resistance of children be due to an immature immune system?

Unlike other respiratory viruses, such as influenza, respiratory syncytial virus, adenovirus, and others, one very intriguing aspect is that the current SARS-CoV-2 pandemic (like with SARS-CoV and MERS) may not cause a more serious illness in immunosuppressed patients in addition to being milder in immature hosts. In a recent letter from a pediatric liver transplantation unit in Bergamo, Italy, D'Antiga 38 noted that there were no cases of ARDS in patients immunosuppressed because of transplantation, chemotherapy, or other immunosuppressive treatments. However, some of these cases were positive for SARS-CoV-2, suggesting that immunosuppressed patients may not be at higher risk of severe pulmonary disease compared with the general population. Nevertheless, this is still purely observational, as is a report of fatal COVID-19 pneumonia in two transplanted patients in China 39. Additionally, another Italian study reported 4% of adults with chronic arthritis diseases under immunosuppressive treatment had suspected or confirmed COVID-19, with no deaths 40.

This brings us to what may prove to be the crucial point in understanding COVID-19 pathophysiology. As in most (if not all) infectious diseases, this disease is not a direct and simple result of the infection, but the consequence of both the presence of the pathogen and its interaction with the patient's immune system. Thus, even if we unveil, as we are indeed unveiling, many characteristics of the virus that contribute to and are coherent with the clinical manifestations and course of COVID-19 without adding to the picture the immune reaction to the virus, we will be missing the target. In addition, by taking into account the immune response, we need to consider that the response in a patient will not be independent of the individual immunological history, where previous infections and momentary immune status will drive the response to one pattern or another and, possibly, to different clinical evolutions of the disease.

Currently, however, we are only beginning to describe the immune response of patients to SARS-CoV-2, and we are unclear about the most effective immune response pattern against the virus. A prospective observation of a 47-year-old female patient with mild-to-moderate COVID-19 showed increased numbers of antibody-secreting cells, follicular helper T cells, activated CD4^+^ T cells and CD8^+^ T cells, as well as antiviral IgM and IgG antibodies in blood before symptomatic recovery 41. This study indicates that early robust adaptive immune responses were elicited against SARS-CoV-2, as should occur in other viral diseases, but we cannot conclude from it whether humoral or cellular responses are more relevant. In contrast, patients who had recovered from SARS showed potent antibody responses specific to the SARS-CoV spike protein with robust neutralizing activity, which persisted at high titers over a three-year follow-up 42. In addition, the IgG level in patients with mild SARS-CoV infection was significantly higher than that in patients with severe infection 43.

If the antibody response is responsible for the severity of COVID-19, we should consider that adults would have come into contact with and have produced antibody responses against several antigens from related viruses throughout their lives on a much larger scale than in children 44. These antibodies could cross-react with SARS-CoV-2 with a low affinity and could induce activation of an inflammatory response, either by local deposition of immune complexes or by binding to Fc receptors present on pulmonary antigen-presenting cells, instead of promoting an effective viral neutralization. In fact, in patients with COVID-19, the innate immune response shows an increase in neutrophil numbers and C-reactive protein (CRP), D-dimer, and IL-6 levels 43,45.

Another possible mechanism through which antibodies could contribute to the severity of the disease is the antibody-dependent enhancement, which is well-described in dengue virus infections 46. This was also, in fact, demonstrated by Yip et al. 47 in SARS-CoV infection of human macrophages *in vitro*. Nevertheless, although murine anti-spike antibodies facilitated human macrophage infection via the Fcγ receptor II (CD32), this resulted in neither SARS-CoV replication nor alteration of pro-inflammatory cytokine/chemokine production or apoptosis-induced ligands by these infected cells. This is relevant because other clinical studies indicate that COVID-19 patients have lymphocytopenia with high levels of several cytokines and chemokines, such as G-CSF, IP-10, MCP-1, MIP-1α, and TNF-α 48,49. Therefore, the increased production of pro-inflammatory cytokines could be the cause of both viral sepsis and damage to tissues or organs, resulting in septic shock, disseminated intravascular coagulation, and multi-organ dysfunction syndrome. These phenomena of a cytokine storm syndrome in COVID-19 are similar to those in hemophagocytic lymphohistiocytosis 50 and in the macrophage activation syndrome associated with systemic-onset juvenile idiopathic arthritis or juvenile systemic lupus erythematosus 51-53, indicating that COVID-19 is, at least in some cases, a disease of immune dysregulation.

Another observation deserves to be highlighted: in the description of the clinical characteristics of coronavirus disease in China, lymphocytopenia (<1.2×10^9^ per liter) was present in only 3.5% of pediatric patients in contrast to 83.2% of the 1,099 patients of all age groups analyzed 6. The characteristically higher numbers of total lymphocytes and their main subpopulations in healthy infants and young children 54 attracts attention and warrants further investigations, although we cannot determine whether this lack of lymphocytopenia is a cause or consequence of a diminished disease severity.

Another hypothesis related to the immune history of patients has been proposed, that is, a “protective” effect of BCG (Bacille Calmette-Guérin) vaccination against tuberculosis, as countries where BCG is compulsorily administrated in the first few days of life, like Brazil, have a seemingly more controlled dissemination of the SARS-CoV-2 virus 55. A recent review discussed the possible non-specific mechanisms of action of BCG or muramyl dipeptide (MDP) against viral infections in animal models and humans 56. The proposed mechanisms were an induction of CD4 and CD8 T-cell responses, mainly of the Th1 and Th17 subtypes, to secondary unrelated viruses 57; an increased functional cross-reactive antibody response 58; and increased production of pro-inflammatory cytokines, such as IL-1β and TNF-α, by epigenetic reprogramming of monocytes and macrophages (“trained immunity”), probably as a consequence of greater activation of CD11b, TLR4, and CD14 on these cells 59,60. Faced with a disease where most pathogenetic mechanisms seem to rely on “excessive” immune responses, these hypotheses would have to be adjusted before one could incorporate them into the picture of the natural history of COVID-19.

As is evident, the pathophysiology of SARS-CoV-2 infection is far from being understood. Most data indicate that it is, in fact, a multisystemic disease and not only a respiratory disorder. Hematologic, cardiac, renal, neurologic, gastrointestinal, and other alterations are being described as parts of a conundrum that needs to be clarified. Understanding the reasons for the consistent observations that immune-immature and some immunosuppressed hosts are spared from severe manifestations could contribute to elucidating COVID-19 aggression mechanisms and indicate pathways to offer better and more efficient treatment to infected patients. Interestingly, after the acceptance of this manuscript, there have been warnings from pediatric associations in Spain, the UK and the USA about cases of children with confirmed COVID-19. These patients developed septic shock and Kawasaki-like features, after initial gastrointestinal manifestations and without flu-like symptoms 61. It is noteworthy that vascular lesions and dysregulated inflammatory responses, which seem to be characteristics of COVID-19 in adults, may also occur in children.

On the last April 27th, Bi et al. 62 published a retrospective cohort study from Shenzhen, China demonstrating that the rate of infection in children below 10 years was similar to the population average, although children are less likely to develop severe symptoms.

## AUTHOR CONTRIBUTIONS

All the authors contributed substantially to the conception and design of the study and in the analysis and interpretation of data. All authors revised the work critically and approved the final version.
